# Developmental delay, coarse facial features, and epilepsy in a patient with *EXT2* gene variants

**DOI:** 10.1002/ccr3.2010

**Published:** 2019-02-19

**Authors:** Aditi Gupta, Sarah A. Ewing, Deborah L. Renaud, Linda Hasadsri, Kimiyo M. Raymond, Eric W. Klee, Ralitza H. Gavrilova

**Affiliations:** ^1^ Center for Individualized Medicine Mayo Clinic Rochester Minnesota; ^2^ Department of Health Sciences Research Mayo Clinic Rochester Minnesota; ^3^ Department of Clinical Genomics Mayo Clinic Rochester Minnesota; ^4^ Department of Pediatric and Adolescent Medicine Mayo Clinic Rochester Minnesota; ^5^ Department of Neurology Mayo Clinic Rochester Minnesota; ^6^ Department of Laboratory Medicine and Pathology Mayo Clinic Rochester Minnesota

**Keywords:** *EXT2*, genetics, *NDST1*, neurology, whole exome sequencing

## Abstract

We report a patient with developmental delay, autism, epilepsy, macrocephaly, facial dysmorphism, gastrointestinal, and behavioral issues due to *EXT2* compound heterozygous likely pathogenic variants. This case report expands the *EXT2* gene mutation database and the clinical spectrum of patients with deficiencies in the heparan sulfate pathway.

## 1 INTRODUCTION

A number of inherited disorders are known to be caused by mutations in genes involved in the biosynthesis of heparan sulfate (HS), a linear polysaccharide that binds protein ligands, forming proteoglycans that are components of cell surfaces and extracellular matrices and are involved in many developmental processes.^1^ *EXT2* (MIM: 608210) and *NDST1* (MIM: 600853) are two such genes critical to the polymerization and processing of heparan sulfate. *EXT2* belongs to the exostosin family of glycosyltransferases involved in the chain elongation step of heparan sulfate biosynthesis,^2^ while *NDST1* encodes the bifunctional enzyme GlcNAc N‐deacetylase/N‐sulfotransferase that modifies the glycosaminoglycan component of heparan sulfate and plays a role in establishing the extent and pattern of HS sulfation.^3^ *EXT2* is implicated in the autosomal dominant disorder multiple Exostoses type 2 (MIM: 133701). Biallelic *EXT2* mutations, however, have also been reported in four siblings born to consanguineous parents, manifesting scoliosis, seizures, and macrocephaly (MIM: 616682) without exostosis, following an autosomal recessive pattern of inheritance.^1^ Recently, two more families have been reported with multiple siblings in each displaying intellectual disability, facial dysmorphisms, and seizures with autosomal recessive *EXT2* variants and this disorder has been renamed with the acronym AREXT2 (autosomal recessive EXT2‐related syndrome).^4^, ^5^ Biallelic pathogenic variants within *NDST1* are implicated in autosomal recessive intellectual disability type 46 (MIM: 616116).

This case report describes a patient with distinctive facial features, developmental delay, autism, and epilepsy, who was found to have two compound heterozygous likely pathogenic variants in *EXT2* and a heterozygous variant of uncertain significance (VUS) within *NDST1* through whole exome sequencing (WES) performed by the Genomics Laboratory at Mayo Clinic.

## 2 CLINICAL REPORT

### 2.1 Clinical description

The patient is a 14‐year‐old female evaluated through the Mayo Clinic Department of Clinical Genomics for developmental delay, autism, epilepsy, and distinctive craniofacial features including macrocephaly, prominent tall forehead, hypertelorism, long hypoplastic philtrum, bilateral preauricular pits, upturned and round broad nasal tip.

She was born to a fraternal twin pregnancy at 36‐5/7 weeks’ gestation via C‐section. Her birth weight was 7 pounds 2 ounces and birth length was 20.5 inches. Apgar scores are unknown, but she required treatment for respiratory difficulties with an oxygen tent and a CPAP for 4 days. She had slightly delayed motor milestones, sat unassisted at 7‐8 months, and walked at 14 months of age. At the age of 2 years, language delay was also evident. Developmental regression of verbal and social skills along with cognitive decline was reported at 27 months. At age 5 years, she was found to be positive for antibodies to neuronal voltage gated potassium channels. She showed some improvement in language and socialization with immunotherapy but did not tolerate this treatment well so it was discontinued. She developed seizures at 10 years of age. She had aggressive behavior toward self and others, rocking stereotypies and sleep difficulty, which improved over time. She underwent surgery for strabismus bilaterally at the age of 5 years. Additional features include sensitive skin prone to acne and gastro‐intestinal problems such as constipation and gastroesophageal reflux.

### 2.2 Laboratory evaluations

The patient had a normal karyotype, array CGH, and molecular analyses for Fragile X, Rett and Angelman/Prader Willi syndromes. Biochemical screening for mucopolysaccharidoses was performed in view of the coarse facial features and revealed low levels of heparan sulfate in dried blood spot (12 nmol/L), serum (4.46 ng/mL), and urine (0.5 mg/mmol creatinine) specimens (Reference ranges given in Table 1). Brain MRI was normal. EEG was abnormal and revealed occipital midline spike and waves consistent with focal seizures.

**Table 1:** Reference range for Heparan Sulfate

Heparan sulfate			
Pediatric reference Range	DBS N = 173 (nmol/L)	Serum N = 268 (ng/mL)	Urine N = 525 (mg/mmol CT)
1%ile	12.0	7.06	0.014
10%ile	16.2	9.30	0.031
50%ile	32.1	15.06	0.067
90%ile	48.9	28.24	0.188
99%ile	96.1	53.89	0.292

### 2.3 Family history

**Figure 1:**
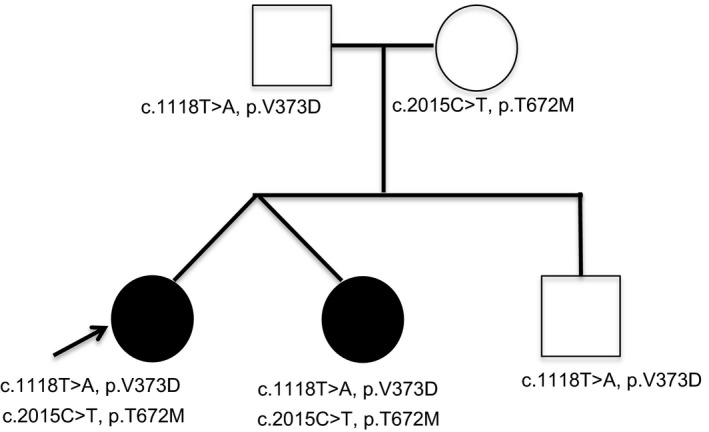
Pedigree of our family showing *EXT2* variants

Her fraternal twin sister has Asperger syndrome (diagnosed at 3 years of age), mild motor delays, and a history of cognitive and speech delays, which had improved over time. There was no other relevant family history, and brother and parents were unaffected (Figure 1).

## 3 METHODS

### 3.1 Editorial policies and ethical considerations

The patient provided written informed consent to research protocol IRB#12‐009346 approved by the Mayo Clinic Institutional Review Board for this study and publication of this paper.

### 3.2 Whole exome sequencing

Whole exome sequencing through the Mayo Clinic Genomics Laboratory was performed on DNA extracted from blood samples from the proband and her unaffected parents. The exome was captured utilizing a custom reagent developed by Mayo Clinic and Agilent Technologies. Sequencing was performed on an Illumina HiSeq 2500 next‐generation sequencing instrument, using HapMap sample NA12878 as an internal control. Paired‐end 101 base‐pair reads were aligned to a modified human reference genome (GRCh37/hg19) using Novoalign (Novocraft Technologies, Malaysia). Sequencing quality was evaluated using FastQC (www.bioinformatics.babraham.ac.uk/projects/fastqc/). All germline variants were jointly called through GATK Haplotype Caller and GenotypeGVCF.^6^ Each variant was annotated using the BioR Toolkit.^7^ Approximately, 97% of the exome is sequenced to a depth of at least 20X. Variants of interest in the patient were confirmed by automated fluorescence dideoxy sequencing and subsequently submitted to ClinVar (https://www.ncbi.nlm.nih.gov/clinvar/) under accession numbers SCV000782709, SCV000782708, and SCV000782711.

### 3.3 In silico analysis tools used

Putative effects of the variants on protein structure and function were predicted using SIFT,^8^ PolyPhen‐2,^9^ Mutation Taster,^10^ M‐CAP,^11^ Predict SNP2,^12^ and CADD.^13^

## 4 RESULTS

Trio WES testing revealed three possibly relevant variants. Two compound heterozygous VUSs (one paternally inherited—c.1118T>A p.(Val373Asp) and a maternally inherited—c.2015C>T p.(Thr672Met)) were identified within the *EXT2* gene. This gene encodes a ubiquitously expressed enzyme involved in heparan sulfate biosynthesis, localized in the Golgi apparatus, but has limited gene‐disease relationships and was considered a gene of uncertain significance (GUS). The paternally inherited variant c.1118T>A (rs371996957) has a frequency of 0.001% in gnomAD (3 out of 246 244 alleles, 0 homozygotes), and the maternally inherited variant c.2015C>T (rs138722406) has a frequency of 0.007% in gnomAD (20 out of 276 874 alleles, 0 homozygotes).^14^ Various in silico tools described in Methods section predicted damaging effects for both variants. For p.Val373Asp, the CADD raw score is 4.1 while the PHRED‐scaled score is 29.3 and for p.Thr672Met, the CADD raw score is 4.4 while the PHRED‐scaled score is 33. Both encoded amino acids are conserved across orthologs, and in both cases the reference and variant amino acids differed with respect to charge, size, and polarity. The first variant (c.1118T>A) was in exon 6 of 14 (NM_000401.3), and on the Exostosin‐like domain while the second variant (c.2015C>T) was located in exon 12 of 14 (NM_000401.3) and in the disulfide rich, substrate binding C‐terminal Exostosin and Transferase domains (Alamut Visual version 2.10 (Interactive Biosoftware, Rouen, France)), signifying the importance of these variants on enzyme activity and function. Additionally, a heterozygous maternally inherited VUS, c.1360C>T p.(Arg454Cys), within *NDST1* was also identified. The *NDST1* alteration c.1360C>T (rs150009231) identified in our patient is a rare variant with a frequency of 0.02% in gnomAD (44 out of 275 364 alleles, 0 homozygotes) and found in a gene predicted to be intolerant to missense and loss of function variants in ExAC.^14^ This variant has conflicting predictions of pathogenicity on various in silico tools as described in the Methods section. Its CADD raw score is 4.2 while the PHRED‐scaled score is 31. The amino acid position is conserved in mammals, and the wild type residue arginine is different from the variant amino acid cysteine in terms of charge, size, and polarity. This variation is present in exon 6 of a total of 15 exons (NM_001543.4) and found in the heparan sulfate N‐deacetylase domain of the protein. However, no second mutation was identified in this patient within this gene, and the deletion/duplication analysis for this gene yielded normal/negative results.

Both the compound heterozygous *EXT2* variants and heterozygous *NDST1* variant were identified in the mildly affected fraternal twin sister.

## 5 DISCUSSION

Review of the sequencing results suggested the *EXT2* variants were of clinical interest and putatively pathogenic relative to the patient's presenting phenotype. The *EXT2* gene encodes a ubiquitously expressed enzyme involved in heparan sulfate biosynthesis, localized in the Golgi apparatus. Heterozygous pathogenic variants within *EXT2* are causative of an autosomal dominant disorder known as multiple Exostoses type 2 in which patients develop multiple benign (ie, non‐malignant) bone tumors called osteochondromas, not present in our patient. Recently, biallelic mutations in *EXT2* have been described as causative for a new disorder named AREXT2 (autosomal recessive EXT2‐related syndrome). This syndrome includes phenotypes of developmental delay, intellectual disability, seizures, scoliosis, and micro/macrocephaly. Patients with this syndrome also have been reported to have coarse facial features, GI‐related issues, and poor speech.^1^, ^4^, ^5^ As illustrated in Table 2, our patient has overlapping features with this disorder, including macrocephaly, coarse facies, long philtrum, hypertelorism, strabismus, gastroesophageal reflux disease, constipation, sensitive skin, development/language delay, seizures, and delayed psychomotor development. However, skeletal issues such as scoliosis and decreased bone density and muscular issues such as hypotonia, observed with this disorder, were not observed in our patient. There is clinical heterogeneity in this disorder, with two of four patients with biallelic *EXT2* alterations noted to have a ventricular septal defect.^1^ The onset of this disorder is reported in infancy, with seizures starting between 2 and 5 years. Our patient's developmental delay was noted during the first 2 years of life and seizures were noted around 10 years of age. Despite some differences, there was significant overlap seen between this clinically variable condition and the patient's phenotype. The two rare *EXT2* variants found in our patient have not been reported clinically in the literature or clinical variant databases (HGMD, OMIM, or ClinVar). However, in silico tools predict they are putatively pathogenic. Notably, the patient's fraternal twin sister who is mildly affected as compared to the proband, with some learning delays and speech concerns, was also found to have the two compound heterozygous *EXT2* variants while the patient's unaffected brother was found to carry only the heterozygous paternally inherited *EXT2* variant (Figure 1).

**Table 2:** Clinical comparison of our patient with previously described patients with AREXT2

Features	Farhan et al 1				El‐Bazzal et al 4		Gentile et al 5		This study	
	Patient 1 II‐1	Patient 2 II‐3	Patient 3 II‐4	Patient 4 II‐5 (deceased)	Patient 1 II‐2	Patient 2 II‐3	Patient 1	Patient 2	Proband	Sister
Sex	M	M	F	M	M	M	M	F	F	F
Age at examination (y)	19	14	11	10	8	6	21	15	14	14
Genotype										
* EXT2* variants	c.260T>G, p.Met87Arg (hom)c.283C>T,p.Arg95Cys (hom)	c.260T>G, p.Met87Arg (hom)c.283C>T, p.Arg95Cys (hom)	c.260T>G, p.Met87Arg (hom)c.283C>T, p.Arg95Cys (hom)	c.260T>G, p.Met87Arg(hom)c.283C>T,p.Arg95Cys (hom)	c.11C>T,p.Ser4Leu(hom)	c.11C>T,p.Ser4Leu(hom)	c.679G>A,p.Asp227Asn(het), c.1823A>G,p.Tyr608Cys (het)	c.679G>A,p.Asp227Asn(het), c.1823A>G,p.Tyr608Cys (het)	c.1118T>A, p.Val373Asp (het),c.2015C>T, p.Thr672Met (het)	c.1118T>A, p.Val373Asp (het),c.2015C>T, p.Thr672Met (het)
* NDST1* variant									c.1360C>T, p.Arg454Cys (het)	c.1360C>T, p.Arg454Cys (het)
Neurological issues										
Developmental Delay	+	+	+	+	++	+++	++	+	++	+
Intellectual disability	+	+	+	+	++	+++	++	+	++	+
Speech delay	+	+	+	+	+	+	+	+	+	+
Autism	−	−	−	−	−	−	+	+	+	−
Seizures	+	+	+	+	+	+	+	+	+	−
Facial dysmorphisms										
Macrocephaly	+	+	+	+	−	−	−	+	+	−
Microcephaly	−	−	−	−	+	+	−	−	−	−
Forehead					Flat	Flat	High	High	Prominent tall	
Hypertelorism	+	+	+	+	−	−	+	+	+	−
Long philtrum	+	+	+	+	−	−	+	+	+	−
Bulbous nose	−	−	−	−	−	−	+	+	+	−
Ear anamolies	−	−	−	−	+	+	−	+	−	−
Other issues										
Exostoses	−	−	−	−	−	−	+	+	−	−
Cardiovascular issues	+	−	+	−	−	−	+	+	−	−
Renal issues	+	+ (Kidney failure)	−	−	−	−	−	+	−	−
Hypotonia	+	+	+	+	+	++	−	+	−	−
Scoliosis	+	+	+	−	−	−	+	+	−	−
Skin issues	Sensitive skin	Sensitive skin	Sensitive skin	Sensitive skin	−	−	−	−	Prone to acne	−
Gastrointestinal issues	Gastroesophageal reflux & associated ulceration, constipation and lately diarrhea	Gastroesophageal reflux, Dysfunctional gastrointestinal motility	+	+	Gastroesophageal reflux	Gastroesophageal reflux, constipation	−	+	Gastroesophageal reflux, constipation	−

+, present; −, absent.

*NDST1* encodes a ubiquitously expressed transmembrane protein residing in the Golgi apparatus, involved in the pathway for formation of heparan sulfate. The gene is implicated with autosomal recessive intellectual disability type 46. This condition has an onset in infancy or early childhood and is characterized by delayed psychomotor development, delayed or absent speech, intellectual disability and hypotonia. Patients in some cases have also been shown to display behavioral abnormalities such as agitation, aggression and sleep disturbances, seizures, and postnatal growth deficiency.^3^, ^15^ The patient had aggressive behavior, intellectual disability, seizures, and sleep disturbance but no evidence of growth retardation. Notably, the mildly affected sister was also found to carry this variant. The discordance in the phenotype of the two sisters might be due to the presence of potassium channel antibodies seen in proband but not her sister. However, as this condition is autosomal recessive and no variant was found in the patient's second allele, this remains a VUS. It is interesting to note that both *EXT2* and *NDST1* are genes that encode enzymes involved in heparan sulfate synthesis. This raises the possibility that the *EXT2* gene variants are possibly causative and the *NDST1* variant has a modifier effect on the patient's clinical characteristics. At this time, there is no sufficient evidence to prove this hypothesis and this remains an open question for future investigation.

## 6 CONCLUSION

In summary, the genetic variants found in *EXT2* are likely pathogenic according to ACMG/AMP guidelines,^16^ based on the collective evidence of familial cosegregation, biochemical analysis, and emerging gene‐phenotype reports in the literature. At this point, we do not have enough evidence to rule in or out a potential contribution of the *NDST1* variant despite the fact both EXT2 and NDST1 proteins are located in the ER‐Golgi, are components of the heparan sulfate biosynthesis pathway, and interact with each other affecting the structure and levels of heparan sulfate,^17^ and this remains a finding of uncertain significance. The findings described above could allow for a better characterization of distinct features of such clinically variable and nonspecific phenotypes and may also provide potential strategies for therapeutic intervention by enzyme replacement therapy or administration of heparan sulfate to the patients.

## CONFLICT OF INTEREST

The authors declare no conflicts of interest exist.

## AUTHOR CONTRIBUTIONS

A.G., L.H., and E.W.K.: summarized the findings and characterized the patient's genomic results. K.M.R.: measured heparan sulfate levels in the patient. R.H.G., D.L.R., and S.A.E.: evaluated the patient clinically, counseled the patient, and obtained consent from the patient. A.G., R.H.G., and E.W.K.: wrote the manuscript with input from all authors.
